# A comparative analysis of safety and short-term efficacy between open and laparoscopic pancreaticoduodenectomy utilizing different drainage techniques

**DOI:** 10.3389/fonc.2026.1775048

**Published:** 2026-04-07

**Authors:** Changjun Liu, Chen Zou, Pengxiang Tang, Hanyu Liu, Weinan Liu, Lian Shen, Kang Chen, Wei Cheng, Bingzhang Tian, Shuo Qi, Bowen Xie

**Affiliations:** 1 Department of Hepatobiliary Surgery, Hunan Provincial People’s Hospital, The First Affiliated Hospital of Hunan Normal University, Changsha, Hunan, China; 2 Department of general surgery, People’s Hospital of Yiyang, Yiyang, Hunan, China; 3 Shi Cheng College, Hunan Normal University, Changsha, Hunan, China; 4 Department of Hepatobiliary Surgery, Loudi Central Hospital of Hunan Province, Loudi, Hunan, China

**Keywords:** clinical efficacy, external drainage, internal drainage, open versus laparoscopic, pancreaticoduodenectomy (PD)

## Abstract

**Purpose:**

To evaluate the safety profiles of open pancreaticoduodenectomy (OPD) and laparoscopic pancreaticoduodenectomy (LPD), and to analyze the respective advantages and disadvantages of various internal and external drainage techniques.

**Methods:**

All 260 patients successfully underwent laparoscopic or open PD. They were divided into laparoscopy and laparotomy groups by surgical approach, and into internal and external drainage groups by drainage method. Four subgroups were then formed: minimally invasive surgical internal drainage (MIS-ID) and minimally invasive surgical external drainage (MIS-ED), open surgical internal surgical drainage (OS-ID) and open surgical external drainage (OS-ED). Perioperative outcomes were compared across all groups and subgroups.

**Results:**

No significant differences were observed between the laparoscopy and laparotomy groups in patient characteristics (*P* > 0.05). Compared to laparotomy group, the laparoscopy group had significantly higher ALB levels on postoperative days 1, 3, and 5, and higher Hb levels on postoperative days 3 and 5 (*P* < 0.05), with no significant differences in intraoperative or other postoperative clinical indicators (*P* > 0.05). No significant differences were found between the MIS-ID and MIS-ED subgroups in perioperative clinical and laboratory parameters (*P* > 0.05). Compared to the OS-ID subgroup, the OS-ED subgroup showed significantly higher ALB levels on postoperative days 3 and 5, and shorter duration of abdominal drainage (*P* < 0.05), with no significant differences in intraoperative or other postoperative clinical indicators (*P* > 0.05).

**Conclusion:**

LPD can be performed safely and effectively without increasing postoperative complications compared to OPD. During LPD, the choice between internal and external drainage does not significantly influence outcomes following PD. Overall, LPD represents a safe and effective surgical approach, demonstrating significant potential for widespread adoption and clinical application.

## Introduction

Pancreaticoduodenectomy (PD), commonly referred to as the Whipple procedure, is a complex and intricate surgical operation (1). It serves as the standard surgical approach for managing pancreatic head cancer and periampullary cancer, and it may also be employed in treating complicated benign conditions at the pancreaticoduodenal junction, such as mass-forming pancreatitis and destructive injuries involving the junction of the pancreatic head and duodenum (2, 3). The standard surgical resection involved in PD encompasses several key anatomical structures: the distal portion of the common bile duct, the pancreatic head, the pyloric region of the stomach, portions of the duodenum, segments of jejunum proximal to these organs, along with adjacent lymph nodes. Furthermore, essential reconstructive procedures—pancreaticojejunostomy, choledochojejunostomy, and gastrojejunostomy—must be performed to restore continuity within the digestive tract (4–6). Consequently, this operation demands an exceptionally high level of technical proficiency from surgeons.

PD entails the resection and reconstruction of multiple vital organs, leading to a relatively high incidence of postoperative complications. The primary surgical complications include pancreatic fistula, biliary fistula, postoperative hemorrhage, delayed gastric emptying, and abdominal infection (3, 7). Among these issues, postoperative pancreatic fistula (POPF) is regarded as one of the most significant challenges in PD. Literature indicates that the incidence of POPF following this procedure ranges from 0% to 30%, exhibiting considerable variability (8, 9). Effectively addressing the occurrence of POPF and implementing appropriate technical interventions are crucial strategies for enhancing patient prognosis.

Open pancreaticoduodenectomy (OPD) is a conventional surgical approach in which surgeons access the abdominal cavity through a large incision to perform the procedure. However, this method inflicts considerable trauma on the patient, leading to a relatively slow postoperative recovery and an increased risk of complications such as incision pain and infection. With the ongoing advancement of minimally invasive medical technology, laparoscopic pancreaticoduodenectomy (LPD) is anticipated to be utilized for more patients. In contrast, LPD is conducted using laparoscopy and specialized instruments, providing enhanced visualization that aids surgeons in accurately identifying blood vessels and critical tissue structures. Furthermore, LPD results in less trauma to the patient, resulting in reduced postoperative pain and expedited recovery times (10, 11). However, it necessitates that surgeons possess extensive experience in laparoscopic procedures along with proficient operational skills.

In PD, both internal and external pancreaticojejunostomy are commonly employed techniques for the drainage of pancreatic fluid. Internal drainage typically involves anastomosing the pancreatic stump to the jejunum, thereby mimicking the physiological pathway for pancreatic fluid discharge. This approach can help maintain intestinal environmental stability and promote recovery of digestive function (12, 13). Conversely, external drainage entails the placement of a drainage tube to facilitate the expulsion of pancreatic fluid from the body. The primary advantage of this method is that it allows for direct monitoring of both the volume and characteristics of drained pancreatic fluid, which aids in timely detection and management of POPF (14, 15). When selecting a method for pancreaticojejunostomy, it is imperative to comprehensively consider multiple factors—including individual patient differences, surgical conditions, and postoperative management—to achieve optimal therapeutic outcomes.

Currently, numerous studies have established that LPD can safely and effectively reduce patient trauma and enhance postoperative recovery when compared to OPD, without significantly increasing the incidence of surgery-related complications (16, 17). However, the efficacy of external drainage versus internal drainage in mitigating the occurrence of POPF remains uncertain. In clinical practice, the choice between external and internal stenting typically depends on the surgeon’s expertise and preference. To further evaluate the safety profiles of OPD compared to LPD, and based on an analysis of the advantages and disadvantages associated with various methods of internal and external drainage, we conducted this study to assess both the safety and short-term surgical efficacy of OPD versus LPD utilizing different drainage techniques. This research aims to provide a theoretical foundation for the clinical implementation of PD.

## Patients and methods

### Study enrollment

This retrospective study gathered clinical data from 260 patients who underwent either OPD or LPD at the Department of Hepatobiliary Surgery, Hunan Provincial People’s Hospital (the First Affiliated Hospital of Hunan Normal University), between January 2022 and June 2024. All participants strictly adhered to the principles outlined in the Declaration of Helsinki, and the study received approval from the Ethics Committee of Hunan Provincial People’s Hospital.

All cases included in this study were conducted by senior hepatobiliary surgeons at our hospital, each possessing over 150 cases of experience in either OPD or LPD. Following meticulous data collection, screening, and thorough review, a total of 260 patients were enrolled in this study, all of whom satisfied the following criteria: (1) They underwent LPD or OPD due to benign or malignant lesions located in the lower common bile duct, papilla of the duodenum, pancreas, or periampullary region; (2) The method for internal or external drainage during pancreaticojejunostomy was determined based on the surgical preferences of different surgeons; (3) The patients or their legal representatives provided informed consent for the procedure. The exclusion criteria were as follows: (1) A history of prior pancreatic surgery or requirements necessitating total pancreatectomy; (2) Severe cardiovascular or respiratory diseases, along with other serious systemic conditions; (3) Incomplete perioperative data.

### Perioperative management

The decision between LPD and OPD—which entails the resection of the pancreatic head, duodenum, a portion of the stomach, and bile duct, along with routine lymph node dissection—is made based on each patient’s individual clinical condition and preoperative imaging data. Digestive tract reconstruction is carried out using the Child procedure, which establishes a pancreaticojejunostomy connecting the pancreatic stump to the jejunum. Conventional pancreatic duct stents are employed for both internal and external drainage of pancreatic fluid; however, the selection of drainage method ultimately rests on the surgeon’s preference. Techniques for performing pancreaticojejunostomy include invagination pancreaticojejunostomy and duct-to-mucosa pancreaticojejunostomy. The selection of technique relies on each surgeon’s professional expertise and personal preferences.

### The definition and classification of POPF

The International Study Group of Pancreatic Fistula (ISGPS) primarily defines and classifies POPF, emphasizing clinical outcomes and the severity of symptoms (18, 19). POPF is defined by an amylase concentration in the drainage fluid that exceeds three times the upper limit of normal serum amylase levels on or after the third postoperative day, which correlates with clinical outcomes. Grading: Grade A: Biochemical leakage characterized solely by elevated amylase levels, without any clinical impact; no specific treatment is required, and it may resolve spontaneously. Grade B: Pancreatic fistula requiring minimal intervention (such as extended drainage or endoscopic examination); this grade may be associated with local infection or inflammation necessitating antibiotic therapy or additional drainage, resulting in a prolonged hospital stay. Grade C: The most severe form of pancreatic fistula, often accompanied by systemic sepsis and organ failure; this condition necessitates admission to an intensive care unit, anti-infective treatment, or surgical intervention, significantly increasing mortality rates.

### Analysis of indicators

The collected clinical data primarily encompass the patient’s age, gender, body mass index (BMI), and underlying medical conditions such as hypertension, diabetes mellitus, and coronary artery disease. Perioperative laboratory assessments include hemoglobin (Hb) levels, liver function indicators—namely total bilirubin (TBIL), albumin (ALB), alanine aminotransferase (ALT), and aspartate aminotransferase (AST)—as well as amylase (AMY) levels and prothrombin time (PT). Preoperatively, the pancreatic duct diameter (PDD) and vascular invasion were evaluated using CT/MR imaging modalities. The pancreatic consistency is classified by the surgeon through palpation as either soft or hard. Intraoperative data predominantly include operation time, hemorrhage, and specifics regarding blood transfusions. Postoperative data encompass the duration of hospital stay following surgery, the time required for the removal of all abdominal drainage tubes, instances of reoperation, and mortality rates within a 90-day period, among other factors. Tumor characteristics are determined based on routine postoperative pathological reports. Postoperative complications are categorized according to the Clavien-Dindo surgical complication grading system while excluding POPF (20, 21). The primary endpoint of this study was the incidence of short-term efficacy—specifically, POPF. Secondary endpoints included the overall postoperative complication rate, laboratory test parameters, and 90-day postoperative mortality rate, among others.

### Statistical analysis

All measurement data are presented as mean ± standard deviation. The differences in measurement data were analyzed using either the independent sample *t*-test or the Mann-Whitney *U* test, depending on appropriateness. Categorical data are expressed as n (%), and comparisons of categorical variables were conducted using the chi-square test or Fisher’s exact test. Statistical analyses were performed using SPSS version 25.0, with a *P* value of less than 0.05 considered statistically significant.

## Results

### Study design

**Figure 1:**
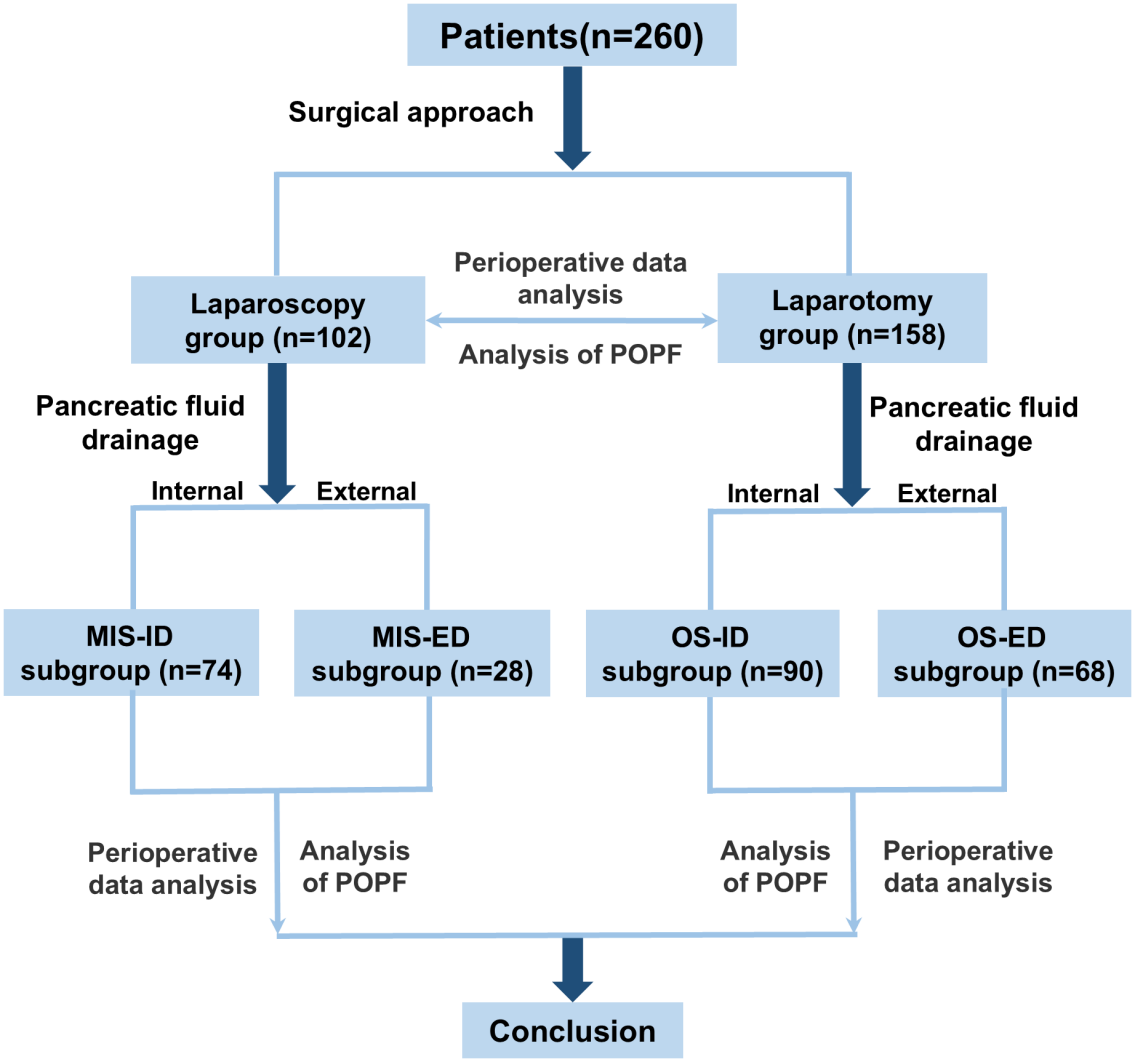
Diagram of study design.

The flowchart depicted in **Figure 1** delineates the design framework of this study. All 260 patients successfully underwent either laparoscopic or open PD. They were categorized into two primary groups: the laparoscopic group and the laparotomy group, based on the surgical techniques employed. Subsequently, each group was further subdivided according to the methods of drainage utilized, resulting in four subgroups: minimally invasive internal drainage (MIS-ID) and minimally invasive external drainage (MIS-ED) for the laparoscopic cohort, as well as open surgical internal drainage (OS-ID) and open surgical external drainage (OS-ED) for those undergoing laparotomy. This classification reflects both internal and external drainage approaches employed during their respective procedures.

### Patients characteristics

The comparative analysis of clinical characteristics between the laparoscopic group and the laparotomy group is summarized in **Table 1**. Among the two groups, variables such as age, BMI, PDD, gender, hypertension, coronary heart disease, diabetes, biliary drainage, vascular invasion, pancreatic consistency, pancreaticojejunostomy type, and tumor classification did not reveal any statistically significant differences (*P* > 0.05). In contrast, pancreatic fluid drainage exhibited statistically significant differences between the two groups (*P* < 0.05).

**Table 1:** Comparative analysis of clinical characteristics between the laparoscopy group and the laparotomy group.

Variate	Total (n=260)	Laparoscopy group (n=102)	Laparotomy group (n=158)	*P* value
Age (y)	60.1±10.9	59.8±10.4	60.3±11.2	0.674
BMI (kg/m^2^)	21.8±3.0	21.8±3.6	21.8±2.6	0.947
PDD (mm)	3.1±2.0	2.9±1.3	3.3±2.4	0.124
Gender				
Male	141(54.2%)	53(52.0%)	88(55.7%)	0.611
Female	119(45.8%)	49(48.0%)	70(44.3%)	
Hypertension				
Yes	85(32.7%)	28(27.5%)	57(36.1%)	0.176
No	175(67.3%)	74(72.5%)	101(63.9%)	
Coronary heart disease				
Yes	16(6.2%)	8(7.8%)	8(5.1%)	0.431
No	244(93.8%)	94(92.2%)	150(94.9%)	
Diabetes				
Yes	40(15.4%)	16(15.7%)	24(15.2%)	0.523
No	220(84.6%)	86(84.3%)	134(84.8%)	
Biliary drainage				
Yes	61(23.5%)	19(18.6%)	42(26.6%)	0.177
No	199(76.5%)	83(81.4%)	116(73.4%)	
Vascular invasion				
Yes	20(7.7%)	5(4.9%)	15(9.5%)	0.235
No	240(92.3%)	97(95.1%)	143(90.5%)	
Pancreatic consistency				
Soft	239(91.9%)	93(91.2%)	146(92.4%)	0.817
Hard	21(8.1%)	9(8.8%)	12(7.6%)	
Pancreatic fluid drainage				
Internal	164(63.1%)	74(72.5%)	90(57.0%)	**0.012**
External	96(36.9%)	28(27.5%)	68(43.0%)	
Pancreaticojejunostomy				
Duct-to-mucosa	177(68.1%)	62(60.8%)	115 (72.8%)	0.056
Invagination	83(31.9%)	40(39.2%)	43(27.2%)	
Tumor type				
Malignant	213(81.9%)	86(84.3%)	127(80.4%)	0.510
Benignant	47(18.1%)	16(15.7%)	31(19.6%)	

Bold values: *P* value < 0.05 indicates statistical significance.

### Comparison and analysis of perioperative data between the laparoscopy group and the laparotomy group

**Figure 2:**
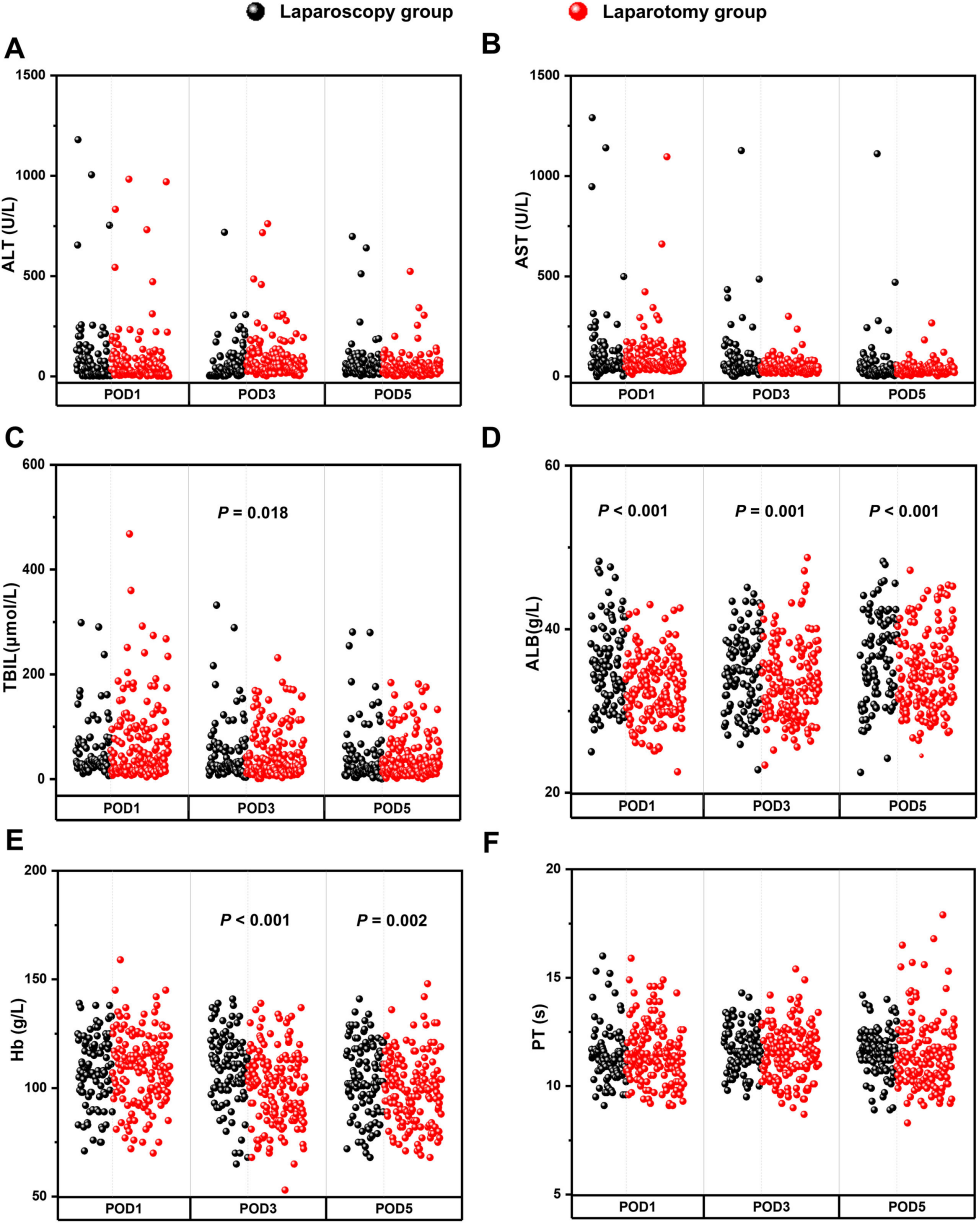
Comparison of postoperative laboratory indexes between the laparoscopy group and the laparotomy group, including POD1, POD3, POD5 value of ALT **(A)**, AST **(B)**, TBIL **(C)**, ALB **(D)**, Hb **(E)** and PT **(F)**.

**Figure 2**; **Table 2** provide a comparative analysis of perioperative data between the laparoscopy group and the laparotomy group. In comparison to the laparotomy group, the laparoscopy group exhibited significantly higher levels of ALB on postoperative days 1, 3, and 5, as well as elevated Hb levels on postoperative days 3 and 5 (*P* < 0.05). However, the laparoscopy group was associated with longer operative times (*P* = 0.001). There were no statistically significant differences observed between the two groups regarding intraoperative hemorrhage, blood transfusion, incidence of POPF, duration until abdominal drainage removal, reoperation rates, or 90-day mortality and postoperative levels of ALT, AST, TBIL, and PT (*P* > 0.05). Furthermore, there were no notable discrepancies observed in the incidence of I - II grade and III - IV grade complications (*P* > 0.05).

**Table 2:** Comparison and analysis of perioperative data between the laparoscopy group and the laparotomy group.

Variate	Laparoscopy group (n=102)	Laparotomy group (n=158)	*P* value
Operation time (min)	450.7 ± 129.1	399.6 ± 102.4	**0.001**
Hemorrhage (mL)	331.5 ± 164.7	290.3 ± 223.9	0.110
Blood transfusion			
Yes	16(15.7%)	25(15.8%)	0.561
No	86(84.3%)	133(84.2%)	
Postoperative pancreatic fistula			
Yes	26(25.5%)	25(15.8%)	0.078
No	76(74.5%)	133(84.2%)	
Postoperative complications			
Grade I-II	10(9.8%)	19(12.3%)	0.688
Grade III-IV	20(19.6%)	20(12.7%)	0.159
Abdominal drainage day (day)	15.9 ± 9.5	13.5 ± 5.6	**0.021**
Postoperative hospital stay (day)	17.1 ± 9.0	16.3 ± 5.4	0.347
Reoperation			
Yes	2(2.0%)	1(0.6%)	0.563
No	100(98.0%)	157(99.4%)	
90-day mortality	0(0.0%)	0(0.0%)	N/A

Bold values: *P* values < 0.05 indicate statistical significance.

### Comparison and analysis of perioperative data between the internal drainage group and the external drainage group

**Figure 3:**
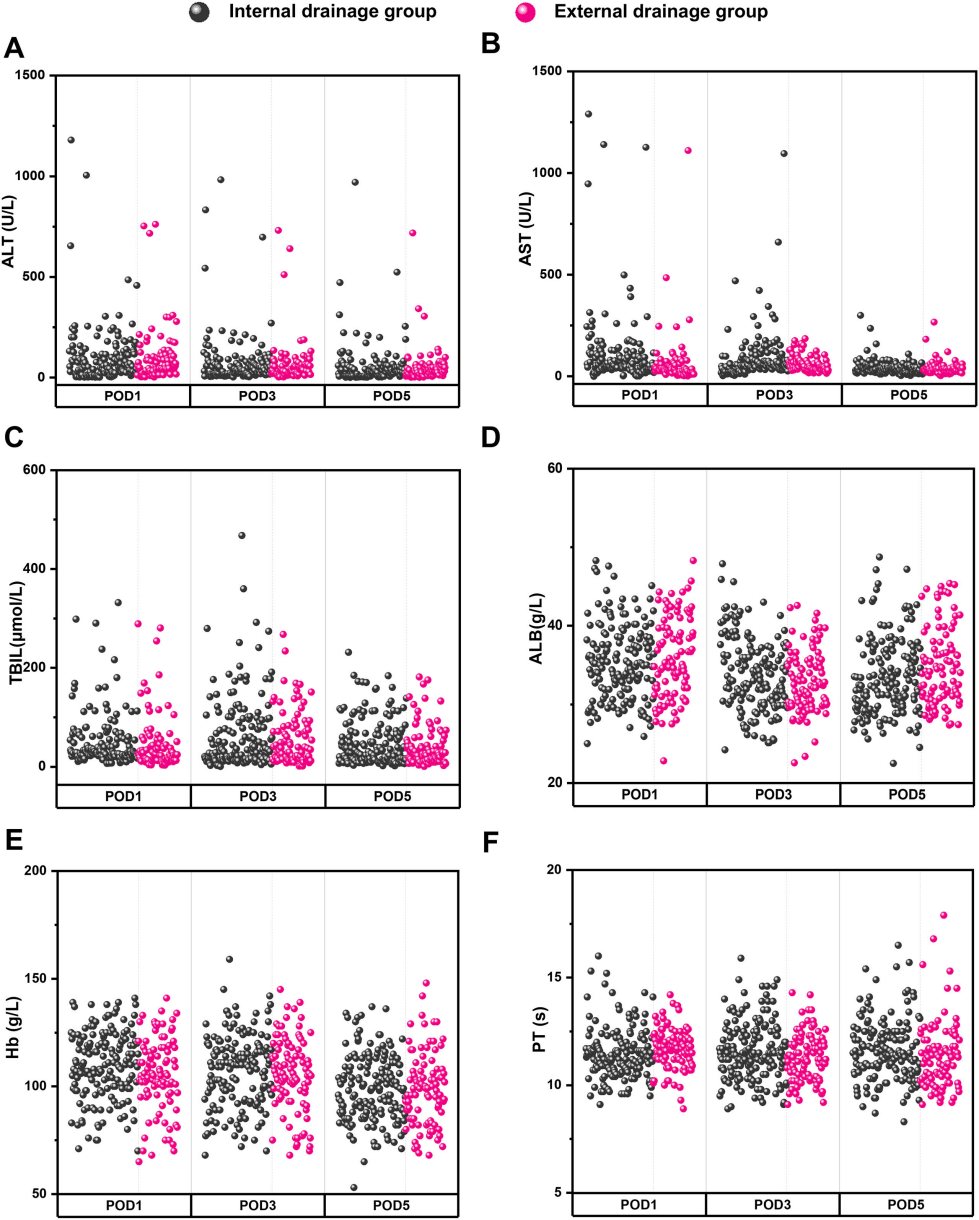
Comparison of postoperative laboratory indexes between the internal drainage group and the external drainage group, including POD1, POD3, POD5 value of ALT **(A)**, AST **(B)**, TBIL **(C)**, ALB **(D)**, Hb **(E)** and PT **(F)**.

The comparison and analysis of perioperative data between the internal drainage group and the external drainage group are presented in **Figure 3**; **Table 3**. No statistically significant differences were observed between the two groups regarding operative time, intraoperative hemorrhage, blood transfusion requirements, incidence of POPF, duration until abdominal drainage removal, length of postoperative hospital stay, reoperation rates, or 90-day mortality (*P* > 0.05). Additionally, there were no significant differences noted in postoperative levels of ALT, AST, TBIL, ALB, Hb and PT (*P* > 0.05). Furthermore, no substantial discrepancies were identified concerning the incidence of complications classified as grade I-II and grade III-IV (*P* > 0.05).

**Table 3:** Comparison and analysis of perioperative data between the internal drainage group and the external drainage group.

Variate	Internal drainage group(n=164)	External drainage group (n=96)	*P* value
Operation time (min)	424.6 ± 117.1	425.8 ± 107.4	0.937
Hemorrhage (mL)	313.8 ± 196.9	294.0 ± 214.4	0.450
Blood transfusion			
Yes	25(15.2%)	16(16.7%)	0.860
No	139(84.8%)	80(83.3%)	
Postoperative pancreatic fistula			
Yes	34(20.7%)	17(17.7%)	0.629
No	130(79.3%)	79(82.3%)	
Postoperative complications			
Grade I-II	16(9.8%)	13(13.5%)	0.415
Grade III-IV	21(12.8%)	19(19.8%)	0.155
Abdominal drainage day (day)	15.0 ± 6.7	13.5 ± 8.6	0.111
Postoperative hospital stay (day)	16.5 ± 6.2	16.8 ± 8.2	0.735
Reoperation			
Yes	2(1.2%)	1(1.0%)	1.000
No	162(98.8%)	95(99.0%)	
90-day mortality	0(0.0%)	0(0.0%)	N/A

### Comparison and analysis of perioperative data between the MIS-ID subgroup and MIS-ED subgroup

**Figure 4:**
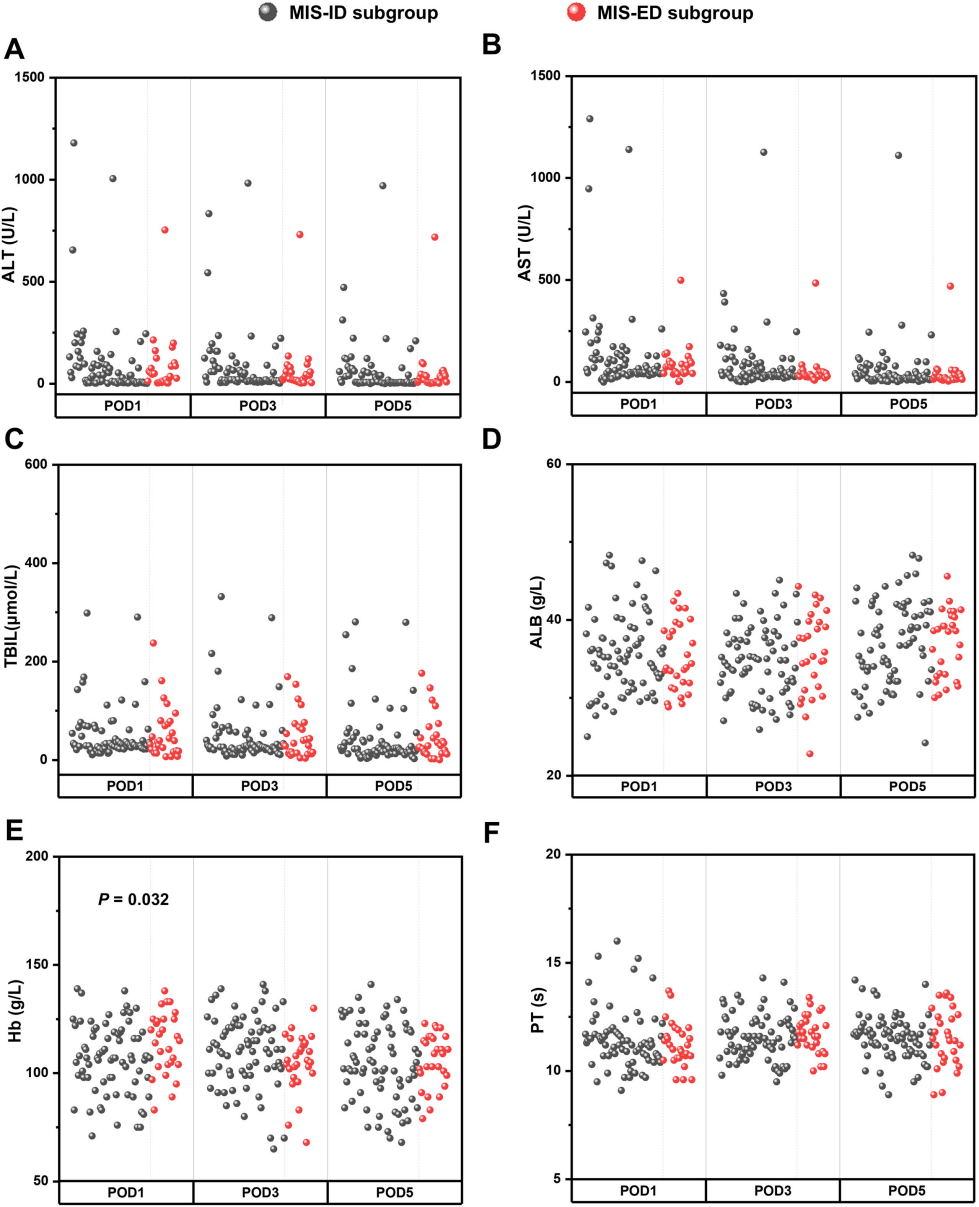
Comparison of postoperative laboratory indexes between the MIS-ID subgroup and MIS-ED subgroup, including POD1, POD3, POD5 value of ALT **(A)**, AST **(B)**, TBIL **(C)**, ALB **(D)**, Hb **(E)** and PT **(F)**.

The comparison and analysis of perioperative data between the MIS-ID subgroup and the MIS-ED subgroup are illustrated in **Figure 4**; **Table 4**. In contrast to the MIS-ID subgroup, patients in the MIS-ED subgroup demonstrated significantly higher levels of Hb on postoperative day 1 (*P* < 0.05). No statistically significant differences were observed between the two groups concerning operative time, intraoperative hemorrhage, blood transfusion, incidence of POPF, duration until abdominal drainage removal, length of postoperative hospital stay, reoperation rates, or 90-day mortality (*P* > 0.05). Additionally, there were no significant differences noted in postoperative levels of ALT, AST, TBIL, ALB, and PT (*P* > 0.05). Furthermore, no notable discrepancies were identified concerning the incidence of I - II grade and III - IV grade complications (*P* > 0.05).

**Table 4:** Comparison and analysis of perioperative data between the MIS-ID subgroup and MIS-ED subgroup.

Variate	MIS-ID subgroup (n=74)	MIS-ED subgroup (n=28)	*P* value
Operation time (min)	443.3 ± 140.6	470.2 ± 91.3	0.262
Hemorrhage (mL)	346.6 ± 171.5	291.8 ± 140.4	0.134
Blood transfusion			
Yes	10(13.5%)	6(21.4%)	0.366
No	64(86.5%)	22(78.6%)	
Postoperative pancreatic fistula			
Yes	17(23.0%)	9(32.1%)	0.445
No	57(77.0%)	19(67.9%)	
Postoperative complications			
Grade I-II	7(9.5%)	3(10.7%)	1.000
Grade III-IV	11(14.9%)	9(32.1%)	0.091
Abdominal drainage days (day)	15.7 ± 7.5	16.3 ± 13.5	0.811
Postoperative hospital stay (day)	16.1 ± 6.8	19.7 ± 12.9	0.073
Reoperation			
Yes	1(1.4%)	1(3.6%)	0.476
No	73(98.6%)	27(96.4%)	
90-day mortality	0(0.0%)	0(0.0%)	N/A

### Comparison and analysis of perioperative data between the OS-ID subgroup and OS-ED subgroup

**Figure 5:**
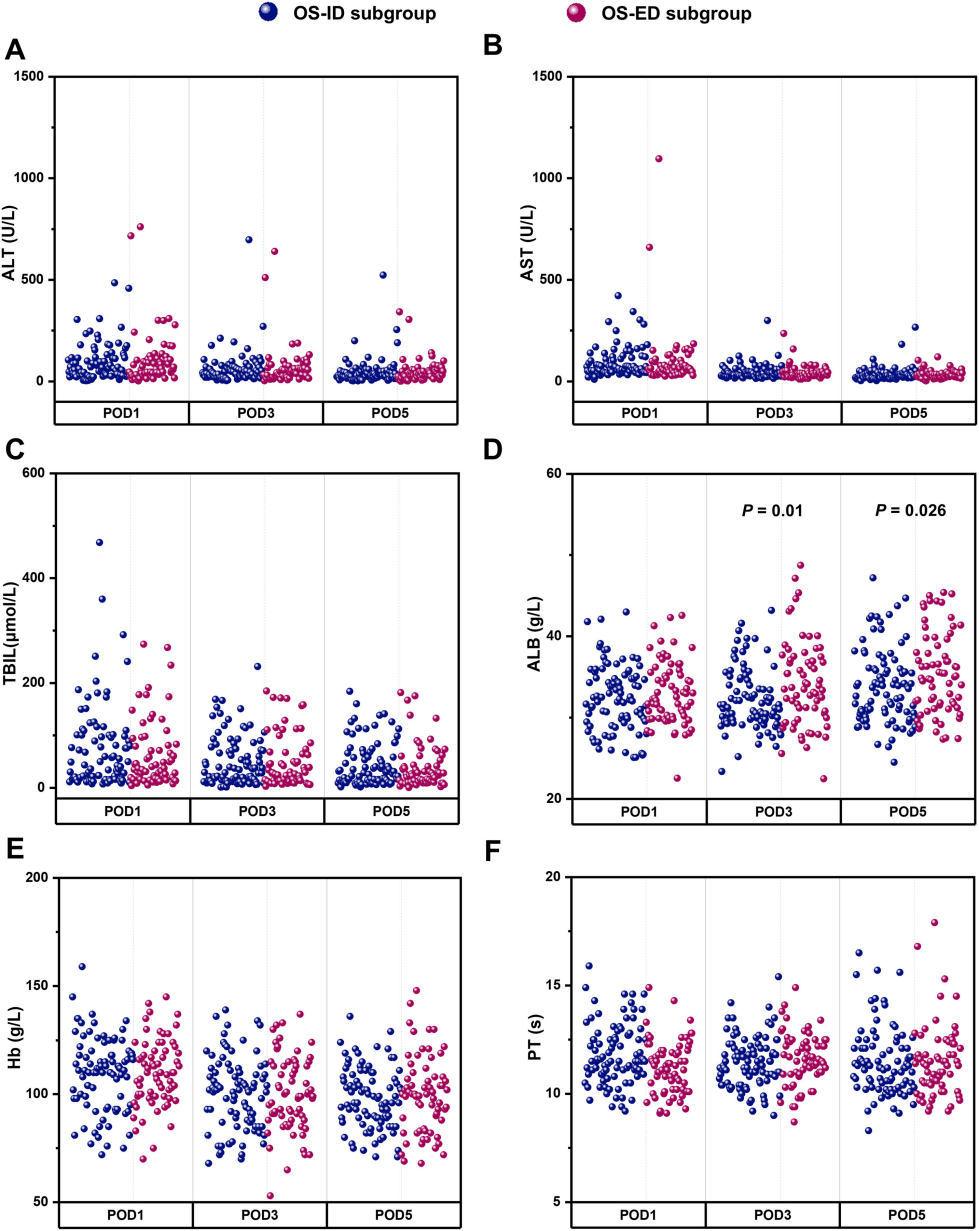
Comparison of postoperative laboratory indexes between the OS-ID subgroup and OS-ED subgroup, including POD1, POD3, POD5 value of ALT **(A)**, AST **(B)**, TBIL **(C)**, ALB **(D)**, Hb **(E)** and PT **(F)**.

The comparison and analysis of perioperative data between the OS-ID subgroup and the OS-ED subgroup are presented in **Figure 5**; **Table 5**. Compared to the OS-ID subgroup, patients in the OS-ED subgroup exhibited significantly higher levels of ALB on postoperative days 3 and 5 (*P* < 0.05). Furthermore, patients in the OS-ED subgroup experienced a shorter duration of abdominal drainage postoperatively (*P* = 0.014). No statistically significant differences were observed between the two groups regarding operative time, intraoperative hemorrhage, blood transfusion, incidence of POPF, length of postoperative hospital stay, reoperation rates, or 90-day mortality (*P* > 0.05). Additionally, there were no significant differences noted in postoperative levels of ALT, AST, TBIL, Hb, and PT (*P* > 0.05). Moreover, no notable discrepancies were identified concerning the incidence of grade I-II and grade III-IV complications (*P* > 0.05).

**Table 5:** Comparison and analysis of perioperative data between the OS-ID subgroup and OS-ED subgroup.

Variate	OS-ID subgroup (n=90)	OS-ED subgroup (n=68)	*P* value
Operation time (min)	404.8 ± 94.0	392.8 ± 113.0	0.467
Hemorrhage (mL)	286.8 ± 212.8	291.9 ± 241.7	0.888
Blood transfusion			
Yes	15(16.7%)	10(14.7%)	0.828
No	75(83.3%)	58(85.3%)	
Postoperative pancreatic fistula			
Yes	17(18.9%)	8(11.8%)	0.274
No	73(81.1%)	60(88.2%)	
Postoperative complications			
Grade I-II	9(10.0%)	10(14.7%)	0.460
Grade III-IV	10(11.1%)	10(14.7%)	0.630
Abdominal drainage day (day)	14.4 ± 5.9	12.1 ± 5.2	**0.014**
Postoperative hospital stay (day)	16.8 ± 5.7	15.4 ± 5.1	0.110
Reoperation			
Yes	1(1.1%)	0(0.0%)	1.000
No	89(98.9%)	68(100.0%)	
90-day mortality	0(0.0%)	0(0.0%)	N/A

Bold values: *P* value < 0.05 indicates statistical significance.

## Discussion

PD is a complex surgical procedure employed to address both benign and malignant tumors situated in the head of the pancreas, ampulla, and surrounding regions. This operation necessitates not only the excision of the pancreas but also involves resection of portions of the duodenum, stomach, common bile duct, and gallbladder. Furthermore, it requires meticulous reconstruction of the digestive tract. The intricacy of this surgery primarily arises from the challenging anatomical structures within this area. The head of the pancreas is encircled by numerous vital blood vessels and nerves; any inadvertent damage could result in severe hemorrhaging or even pose life-threatening risks (20, 21). This complicates delineating clear margins for surgical resection and heightens operational difficulty. Consequently, there are exceptionally high technical demands placed on the surgical team. It is imperative that surgeons possess exceptional operative skills coupled with extensive clinical experience. Moreover, seamless collaboration among anesthesiology personnel, nursing staff, and other medical professionals is essential to effectively manage various risks and complications that may arise during surgery—ultimately ensuring both procedural success and patient safety.

With ongoing advancements in surgical techniques, enhancements in surgical instruments, and optimization of perioperative care protocols, the mortality rate for patients undergoing PD has decreased to below 3% (22). However, postoperative complications continue to occur at an incidence as high as 30% (23, 24). Common complications include POPF, biliary fistulae, delayed gastric emptying, and intra-abdominal hemorrhage. Among these complications, POPF represents one of the most severe challenges; it can significantly impair patients’ quality of life and present substantial clinical difficulties (25). In this study, the overall incidence of POPF was 19.6%, occurring in 51 patients; among these, 7 developed Grade C POPF, while the remaining 44 had Grade B POPF. In the LPD group, 4 patients developed Grade C POPF: two underwent interventional hemostasis, and the other two required reoperation. In the OPD group, 3 patients developed Grade C POPF postoperatively—two received interventional hemostasis, and one underwent reoperation. Consequently, addressing this issue remains one of the most pressing concerns during the perioperative period following PD.

The procedure of PD primarily encompasses two surgical approaches: laparoscopic and open techniques. Compared to OPD, LPD offers significant advantages in terms of minimally invasive techniques. By employing smaller incisions, this approach significantly reduces tissue trauma, lowers the risk of intraoperative bleeding, and results in diminished postoperative pain (26, 27). Conversely, open surgery provides direct visualization and a larger operating field, making it particularly suitable for complex or extensive lesions; however, it is more traumatic and associated with slower postoperative recovery as well as an elevated risk of infection (28, 29). In this study, we evaluated the safety and short-term efficacy of both LPD and OPD. Although the laparoscopy group had a slightly longer operative time (450.7 ± 129.1 minutes) compared with the laparotomy group (399.6 ± 102.4 minutes), no statistically significant differences were observed between the two groups in terms of perioperative blood loss, blood transfusion requirements, length of postoperative hospital stay, overall complication rates, or reoperation rates. These findings collectively support the favorable safety profile of LPD. Consequently, clinical decision-making should be guided by patient-specific factors such as overall health status and tumor characteristics alongside the expertise available within the medical team to optimize therapeutic outcomes.

Pancreatic fluid secreted by the pancreas can be effectively drained through stents to mitigate the risk of POPF, which can be categorized into internal and external drainage methods. Internal drainage is considered more physiological, as it minimizes pancreatic fluid loss and reduces the likelihood of electrolyte imbalances (30, 31). However, if gastrointestinal function does not recover promptly following surgery, there is a potential for pancreatic fluid accumulation, thereby increasing the risk of POPF. Conversely, external drainage allows for complete evacuation of pancreatic fluid, preventing early accumulation and alleviating tension on the anastomosis while facilitating monitoring of pancreatic fluid conditions. Nonetheless, this approach may result in the loss of digestive fluids, potentially leading to electrolyte imbalances and posing greater challenges in postoperative care (32, 33). Some studies have indicated that there are no significant differences in short-term efficacy between these two methods post-surgery; thus far, no definitive conclusion has been reached regarding whether to select internal or external drainage during PD (34, 35). In this study, no significant differences were observed between the internal drainage and external drainage groups with respect to perioperative safety or short-term efficacy. Subsequently, in the minimally invasive surgery subgroup, there were also no significant differences between the internal and external drainage groups in terms of perioperative safety or short-term efficacy. In contrast, within the open surgery subgroup, patients who received external drainage exhibited higher postoperative albumin levels and shorter durations to abdominal drain removal. However, this finding has not been corrected for multiple comparisons and therefore cannot be interpreted as conclusive evidence. Given the potential over-conservatism introduced by such corrections—particularly in exploratory research—we have opted not to apply them, thereby preserving statistical power to detect plausible signals. Nevertheless, these analyses do not support firm conclusions and should be viewed strictly as preliminary evidence to inform hypothesis generation and guide future clinical investigation. In clinical practice, decisions regarding drainage should be made comprehensively based on factors such as surgeon experience, available equipment conditions, and individual patient characteristics to effectively minimize the risk of POPF while promoting optimal postoperative recovery.

This study initially confirmed the safety and efficacy of LPD, and further evaluated the safety and effectiveness of internal drainage versus external drainage under both laparoscopic and open surgical approaches. These findings provide valuable clinical reference for selecting appropriate surgical and drainage techniques in PD. However, this study has several limitations. As a single-center retrospective study, this investigation lacks multicenter validation and large-scale sample data, and its relatively small sample size may compromise the robustness of the findings. Moreover, the selection of surgical and drainage approaches was neither randomized nor guided by predefined clinical criteria; instead, it was determined solely by the operating surgeon on the basis of clinical experience, patient-specific anatomical features, and intraoperative assessment—potentially introducing selection bias. Nevertheless, as all reported findings are exploratory in nature rather than confirmatory, they should be interpreted as hypothesis-generating and serve only as preliminary references for clinical practice. In addition, the subgroup analyses reported in this article are exploratory and were conducted without adjustment for multiple comparisons. Consequently, the findings should be interpreted solely as hypothesis-generating and must not be regarded as definitive or confirmatory evidence. Given the risk of excessive conservatism—particularly in exploratory research—associated with standard multiple-comparison corrections, we deliberately refrained from applying such adjustments to preserve statistical power and enhance sensitivity for detecting potential signals. In the future, we plan to conduct a multicenter randomized controlled study (RCTs) to systematically investigate and analyze the association between laparoscopic and open PD and POPF across different regions and institutions, particularly under various drainage methods. This effort aims to generate higher-level evidence to better inform clinical decision-making regarding surgical and drainage strategies for PD.

## Conclusion

This study initially demonstrated that LPD can be safely and effectively performed without increasing the incidence of postoperative complications compared to OPD. Furthermore, the findings indicate that different methods of internal and external drainage during LPD do not significantly affect clinical or laboratory outcomes in patients. Overall, LPD represents a safe and effective surgical approach, demonstrating significant potential for widespread adoption and clinical application.

## Data Availability

The raw data supporting the conclusions of this article will be made available by the authors, without undue reservation.
